# Factors associated with indeterminate QuantiFERON-TB Gold Plus Test results during the COVID-19 pandemic

**DOI:** 10.1371/journal.pone.0326615

**Published:** 2025-07-21

**Authors:** Carla M. Román-Montes, Karla M. Tamez-Torres, Guillermo A. Guaracha-Basañez, Alberto Ordinola-Navarro, Marco Antonio Ortiz-Bustamante, Sandra Rajme-López, Bernardo A. Martínez-Guerra, Anabel Ordaz-Vázquez, José Sifuentes-Osornio, Alfredo Ponce-de-León, Fernanda González-Lara, Miriam Bobadilla-Del-Valle

**Affiliations:** 1 Infectious Diseases Department, Instituto Nacional de Ciencias Médicas y Nutrición Salvador Zubirán, Mexico City, Mexico; 2 Immunology and Rheumatology Department, Instituto Nacional de Ciencias Médicas y Nutrición Salvador Zubirán, Mexico City, Mexico; 3 Clinical Microbiology Laboratory, Instituto Nacional de Ciencias Médicas y Nutrición Salvador Zubirán, Mexico City, Mexico; 4 General Direction, Instituto Nacional de Ciencias Médicas y Nutrición Salvador Zubirán, Mexico City, Mexico; U.S. Food and Drug Administration, UNITED STATES OF AMERICA

## Abstract

**Objective:**

Indeterminate QuantiFERON (QFT) results challenge clinical decision-making and often necessitate repeat testing. This study aimed to assess the prevalence of indeterminate QFT results and identify associated factors.

**Method:**

We compared patients with indeterminate QFT results to a 1:1 randomly selected sample of patients with determinate results from a tertiary care center in Mexico City between March 2020 and December 2022.

**Results:**

Among 4,557 QFT®-Plus tests performed during the study period, 10% yielded indeterminate results. A total of 352 cases with indeterminate results and 352 with determinate results were analyzed. In 96% of cases, indeterminate results were attributed to a low mitogen response. No significant differences were observed in age, sex, or comorbidities between groups. Multiple regression analysis identified the following factors as significantly associated with an indeterminate QFT®-Plus result: severe COVID-19 (OR 3.9, 95% CI 2.5–6.2, p < 0.001), pharmacological immunosuppression (OR 1.7, 95% CI 1.2–2.4, p = 0.004), severe lymphopenia (OR 1.7, 95% CI 1.1–2.7, p = 0.02), anemia (OR 1.9, 95% CI 1.3–2.8, p = 0.001) and hospitalization (non–COVID–19) (OR 3.9, 95% CI 2.6–5.9, p < 0.001).

**Conclusion:**

The prevalence of indeterminate QFT®-Plus test results was 10%, which is significant, particularly among patients with COVID-19. Indeterminate results were linked to immunosuppression and markers of disease severity. These findings suggest that it may be advisable to postpone QFT®-Plus testing until the clinical condition of patients improves.

## Introduction

Recently, the definition of tuberculosis infection (TBI) has been changed and refers to a state of persistent immune response to previously acquired *Mycobacterium tuberculosis* (MTB) antigens without evidence of symptoms and signs of tuberculosis, previously termed “latent TB” [1,2]. TBI affects one-third of the world’s population, 10% of whom will develop active disease during their lifetime, mainly within the first five years of infection. TBI preventive treatment has been shown to reduce the risk of progression to active tuberculous disease (TB) by 60–90% [3].

Since 2010, the Centers for Disease Control and Prevention updated the recommendations for TBI screening [4]. The use of an interferon-gamma release assay (IGRA) is expected to be more specific than a tuberculin skin test (TST) because the antigens used, ESAT-6 (6 kDa early secreted antigenic target protein) and CFP10 (10 kDa culture filtrate protein) are relatively specific to *M. tuberculosis* complex and should produce fewer false-positive tests [5,6]. The European League Against Rheumatism (EULAR) recommends IGRA over TST due to an observed reduced effect by immunosuppressive drugs, glucocorticoids, or disease-modifying drugs in the former [7]. The treatment of TBI primarily targets high-risk patients, including those who are immunosuppressed, such as organ transplant recipients, rheumatic patients, cancer patients, and individuals living with HIV. Pharmacological immunosuppression included patients taking 15 mg per day of prednisone or its equivalent for one month or longer.

Additionally, TNF-alpha antagonists and other biologic medications are included in this definition. Recently, the relevance of a TB-COVID coinfection has been brought to light [8]. During the COVID-19 pandemic, patients were routinely treated with tocilizumab, baricitinib, or glucocorticoids, making screening for TBI necessary. Hence, identifying high-risk patients and starting TB preventive treatment or close vigilance is of cardinal relevance in managing COVID-19 and post-COVID patients [9].

Currently available in Mexico is the QuantiFERON-TB Gold Plus (QFT®-Plus, Cellestis/Qiagen, Carnegie, Australia); the test includes a Tb1 antigen tube containing long peptides that elicit TCD4 responses, and Tb2, containing ESAT-6 and CFP10. Tb2 binds to the classes I and II major histocompatibility complex (MHC), thus stimulating TCD4 and TCD8 cells, enhancing the sensitivity. Tb1 only binds to Class II MHC peptides, which stimulate only TCD4. Also, QFT-Plus does not use Tb7.7, which can cross-react with non-tuberculous mycobacteria and BCG vaccine strains, thus enhancing specificity [10]. The test provides a positive, negative, or indeterminate result. The causes of an indeterminate result are the lack of response to the mitogen (positive control with phytohemagglutinin) or the high response to the negative control (Nil tube).

Clinical associations with a low mitogen response include older age, hypoalbuminemia, glucocorticoid use, other immunosuppressors, anemia, hospitalization, and patients with chronic comorbidities who become critically ill. The reported prevalence of indeterminate results ranges from 5 to 19% [11–14]. Immunosuppressive agents such as glucocorticoids or anti-TNF-α inhibitors can potentially reduce the production of IFN-γ, IL-1, and TNF-α by T lymphocytes, decreasing the response to mitogen and leading to an indeterminate result. During the COVID-19 pandemic, an increase in indeterminate QFT®-Plus results was reported, with rates up to 35% [15–17]. The rise in immunosuppressive therapies during COVID-19 has increased the demand for QFT®-Plus tests [15]. The results of studies involving COVID-19 patients and some at-risk patients are heterogeneous. Recent studies have highlighted the need for more information on the factors associated with indeterminate QFT [14,18].

This study aims to describe the prevalence of indeterminate QFT®-Plus test results and the factors associated with them during the COVID-19 pandemic.

## Materials and methods

### Study design

We conducted a retrospective, analytical, cross-sectional study. Data collection started on May 2nd, 2023, and included all QFT®-Plus tests performed between March 2020 and December 2022 from a tertiary care center in Mexico City. It is relevant that in March 2020, the hospital was converted into a COVID-19 center, and in July 2020, it transitioned to a hybrid care model.

Participants. Out of the 4,557 tests performed at the center, 10% (n = 463) were indeterminate results. After excluding duplicates and missing data, 352 of these were analyzable. From the remaining 90% (n = 4,094) that had determinate results (either positive or negative), we randomly selected a sample with a 1:1 ratio to compare characteristics with indeterminate results and identify associated factors. Only one test result was considered per patient. The authors did not have access to information that could identify individual participants.

Demographic and clinical data were extracted from the hospital’s electronic medical records. QFT®-Plus testing was ordered by treating physicians as part of their standard practice. For patients with severe COVID-19, local recommendations included QFT®-Plus screening, as corticosteroids and immunosuppressants were considered standard treatment for COVID-19.

### QFT®-plus assay

The test was performed following the manufacturer´s instructions. The samples were collected into lithium-heparin tubes. The version used was the enzyme-linked immunosorbent assay (ELISA). The procedure involves two stages: blood Collection, incubation, plasma harvesting, and the ELISA test.

Sample processing and testing were conducted strictly according to the manufacturer’s instructions in the test insert, ensuring compliance at each step of the procedure and the interpretation of results. Additionally, the laboratory followed an internal Standard Operating Procedure (SOP), local name IO-MICL-325, to ensure standardized handling and execution by trained personnel. Sample recognition was documented manually using a worksheet that recorded the sample number, run number, processing date, and batch number of the kit used. The following critical steps were carefully controlled: (1) collection of exactly 1 mL of whole blood in each QFT-Plus Blood Collection Tube; (2) immediate homogenization of the sample by shaking each tube ten times with sufficient force to coat the inner surface and dissolve the antigens; (3) incubation of tubes in a vertical position at 37°C ± 1°C for 16–24 hours; (4) centrifugation of the tubes post-incubation at 2,000–3,000 × g for 15 minutes to facilitate plasma separation; (5) plasma harvesting was performed without disturbing the gel layer and avoiding pipetting or mixing of the plasma beforehand.

Before performing the ELISA, all plasma samples and reagents—except the Conjugate 100 × concentrate—were equilibrated to room temperature (22°C ± 5°C) for at least 60 minutes. Standard dilutions were carefully prepared to generate the calibration curve, with reconstitution of the standard to achieve a concentration of 8.0 IU/mL. The lyophilized Conjugate 100 × concentrate was reconstituted with 0.3 mL of deionized water and mixed gently to prevent frothing and ensure complete solubilization. Fifty µL of freshly prepared working-strength conjugate and 50 µL of test plasma were added to each ELISA well, followed by incubation at room temperature in the dark for 120 ± 5 minutes. After washing the plate manually six times (removing liquid into a waste tray and blotting on fresh absorbent towels between each wash), 100 µL of enzyme substrate was added, and the plate was shaken and incubated for 30 minutes in the dark. Optical density (OD) was measured within 5 minutes using a microplate reader with a 450 nm filter and a 620 nm reference filter.

Test interpretation was performed using QFT-Plus software, which automatically conducts quality control checks, generates the standard curve, and provides the result.

A test was considered positive when TB1-Nil or TB2-Nil was ≥ 0.35 IU/mL and ≥ 25% over Nil. Indeterminate results were identified in two main scenarios: a low mitogen response (< 0.5 IU/mL) or a high Nil value (> 8.0 IU/mL). As indicated by the manufacturer, low response to mitogen (<0.5 IU/ml) may occur with low lymphocyte counts, reduced lymphocyte activity due to improper specimen handling, problems during filling/mixing of the mitogen tube, or inability of the patient’s lymphocytes to generate IFN-γ. Elevated levels of IFN-γ in the Nil sample may occur with heterophile antibodies or intrinsic IFN-γ secretion, but the test adjusts for this “background activity”. Elevated IFN-γ levels in the Nil tube (>0.8 IU/mL) were the least frequent cause of indeterminate results.

Washing is a critical step in the ELISA workflow. It is designed to remove any unbound components, such as antibodies, antigens, or excess reagents, from the wells. This step is essential to ensure the result accurately reflects only the specific antigen-antibody interactions being measured.

### Definitions

TBI was defined as a positive IGRA test without signs or symptoms of active tuberculosis.

Autoimmune diseases included patients with organ-specific conditions requiring immunosuppressive therapy, such as inflammatory bowel disease, autoimmune hepatitis, and myasthenia gravis. Rheumatic diseases encompass systemic autoimmune conditions, including systemic lupus erythematosus and rheumatoid arthritis. Advanced HIV disease (AHIVD) was defined as a CD4 cell count of <200 cells/mm³.

Blood samples for biochemical testing were collected 48 hours before or after obtaining the QFT®-Plus sample. Biochemical test definitions included leukopenia, defined as a total leukocyte count of <4,500 × 10³/L; lymphopenia, defined as a lymphocyte count of <1,500 × 10³/L; and severe lymphopenia, defined as a lymphocyte count of <500 × 10³/L. C-reactive protein (CRP) cutoff was > 1 mg/dL, while hypoalbuminemia was defined as a serum albumin level of <3.5 g/dL.

Sample size. Here is the process of calculating the required sample size to estimate the prevalence of indeterminate QFT test results, using a reported prevalence of 4.8% as a reference [19] To calculate a population proportion with a specified level of confidence of 95% and a margin of error of 5%, the following formula is used $n=\frac{z^{2\;}\cdot p\cdot (1-p)}{d^{2}}$, where n = required sample size, Z = Z-score corresponding to the desired confidence level, p = expected prevalence, and d margin of error (precision). The sample size substituting the values into the formula is: $n=\frac{0.1752}{0.0025}=70$. The required sample size for estimating the prevalence of indeterminate QFT results is approximately 70 participants.

### Statistical analysis

All analyses were performed using STATA 14v (Stata Corp, College Station, TX). Descriptive analyses were conducted using numbers and proportions, means with standard deviations (SD), or medians with interquartile ranges (IQR), as appropriate. The distribution of variables was determined with the Shapiro-Wilk test.

Based on data distribution, group comparisons were performed using the Chi-squared (Chi²) test, Fisher’s exact test, and the student’s t-test or Wilcoxon rank-sum test. Variables with a p-value <0.2 in the bivariate analysis and those with biological plausibility were included in a multiple logistic regression model to identify factors associated with an indeterminate QFT®-Plus result. Possible collinearity was assessed and ruled out based on the variance inflation factor (VIF), which was not significant. The model discrimination was also evaluated with an area under the ROC curve, and the goodness of fit was assessed using the Hosmer-Lemeshow test. The 95% confidence intervals (95% CI) were reported, and a p-value <0.05 was considered statistically significant.

### Ethics

The Local Institutional Review Board approved the study. Due to its retrospective nature, written informed consent was waived.

## Results

Out of 4557 QTF®-Plus tests, 10% (n = 463) were indeterminate. After data curation, 352 indeterminate results were compared with 352 randomly determined results.

The primary cause of indeterminate results was a low mitogen response, accounting for 96% (n = 337) of cases, while 4% (n = 15) resulted from a high response in the null control. Notably, among these cases, only two patients had COVID-19, indicating that 98% (n = 95/97) of indeterminate results in COVID-19 patients were due to a low mitogen response.

Among patients with determinate results, 16% (n = 55/352) tested positive, while 84% (n = 298/352) were negative. None of the patients with a positive QTF®-Plus result had active tuberculosis, and 27% (n = 15/55) reported receiving tuberculosis preventive therapy with isoniazid or rifampicin after the QFT®-Plus test.

Table 1 shows demographic and clinical data. Among patients with indeterminate QFT®-Plus results, 50% were female (n = 177/352). The median age of these patients was 51 years (IQR 36−63), and 19% (n = 68 out of 352) were older than 65 years. Among the 352 indeterminate results, 28% (n = 97/352) were COVID-19 patients.

**Table 1:** Demographic and baseline characteristics between the indeterminate vs. determinate QFT®-Plus test.

Basal characteristics	Overall n = 704(%)	Indeterminate n = 352(%)	Determinate n = 352(%)	*p* value
Female	362(51)	177(50)	185(53)	0.55
Age, median (IQR)	51(37-62)	51(36-63)	51(38-61.5)	0.62
Age > 65 years	122(17)	68(19)	54(15)	0.16
Health-care worker	36(5)	18(5)	18(5)	1.0
*Comorbidities*				
Obesity	121(17)	52(15)	69(20)	0.09
Low BMI (<18.5 kg/m2)	56(8)	37(10.5)	19(5)	0.01
Diabetes	109(15.5)	47(13)	62(18)	0.12
Rheumatic diseases	145(21)	78(22)	67(19)	0.30
Other autoimmune diseases	36(5)	18(5)	18(5)	1.0
Hematological malignancy	83(12)	44(12.5)	39(11)	0.55
Solid neoplasm	35(5)	22(6)	13(4)	0.12
Chronic liver disease	97(14)	63(18)	34(10)	0.002
Chronic kidney disease	88(12.5)	35(10)	53(15)	0.04
Advanced HIV disease	33(29)	22(73)	11(20)	<0.01
Organ Transplantation	32(4.5)	16(4.5)	16(4.5)	1.0
Pharmacological immunosuppression	312(44)	189(54)	123(35)	<0.01
Hospitalization (non-COVID-19)	266(38)	185(53)	81(23)	<0.01
COVID-19	150(21)	97(28)	53(15)	<0.01
Corticosteroids for COVID-19	98/150 (65)	68/97(70)	30/53(57)	0.08
*Biochemical parameters*				
Leukopenia	159(23)	93(26)	66(19)	0.01
Lymphopenia	325(46)	203(58)	122(35)	<0.01
Severe Lymphopenia	147(21)	106(30)	41(12)	<0.01
Anemia	351(50)	233(66)	118(33.5)	<0.001
Higher CRP levels	352 (50)	334(52)	18(29)	<0.001
Hypoalbuminemia	236(33.5)	170(48)	66(19)	<0.001

Note: IQR, interquartile range; BMI, body mass index; HIV, human immunodeficiency virus; CRP, C-reactive protein.

In the bivariate analysis, patients with indeterminate QFT®-Plus results were more likely to have: a low BMI (10.5% vs. 5%, p = 0.01), chronic liver disease (18% vs. 10%, p = 0.002), advanced HIV disease (73% vs. 20%, p < 0.01), leukopenia (26% vs. 19%, p = 0.01), severe lymphopenia (30% vs. 12%, p < 0.01), hypoalbuminemia (48% vs. 19%, p < 0.001). They had a higher median C-reactive protein level (7.7 mg/dL vs. 1.9 mg/dL, p < 0.01).

Patients with immunosuppression were 44% (n = 312) with a difference between indeterminate (n = 189) vs. determinate (n = 123) QFT®-Plus results, p < 0.01. The type of immunosuppressors were azathioprine 11% (n = 20) vs 7% (n = 9) p = 0.045, mycophenolate 22% (n = 43 vs. 11%(n = 14), p=<0.01, tacrolimus 12% (n = 22) vs. 2% (n = 3) p=<0.01, and rituximab 6% (n = 11) vs. 2% (n = 3), p = 0.05. Methotrexate 6% (n = 11) vs. 7% (n = 9) and leflunomide 7% (n = 3) vs. 1% (n = 1) did not have a statistical difference.

In multivariate logistic regression analysis, adjusting for age and sex, the following factors were independently associated with an indeterminate QFT®-Plus result (Table 2): current pharmacological immunosuppression, severe COVID-19, hospitalization for any cause, severe lymphopenia, and anemia.

**Table 2:** Multivariate logistic regression analysis for factors associated with indeterminate QFT®-Plus result.

	aOR	95% CI	*p* value
Pharmacological immunosuppression	1.7	1.2-2.4	0.004
Hospitalization (non-COVID-19)	3.9	2.6-5.9	<0.001
Severe COVID-19	3.9	2.5-6.2	<0.001
Severe lymphopenia	1.7	1.1-2.7	0.02
Anemia	1.9	1.3-2.8	0.001
Hypoalbuminemia	2.4	1.6-3.6	<0.001

Note: aOR, adjusted Odds-ratio; 95% Confidence Interval. Possible collinearity was assessed and ruled out based on the variance inflation factor, which was not significant. Additionally, the model demonstrated good discrimination, as indicated by an area under the ROC curve of 0.795. The model’s goodness of fit, evaluated using the Hosmer-Lemeshow test, yielded a p-value of 0.40, suggesting a good fit.

### COVID-19 patients

Of the 150 patients with severe COVID-19, 65% (n = 97/150) had an indeterminate QFT®-Plus test result. COVID-19 patients with an indeterminate result were significantly older [61 (IQR 48–72) vs. 54 (IQR 48–64) years, p = 0.02], had their blood sample collected later in the course of infection [median of 9 days [(IQR 6–14) vs. 4 (IQR 2–7), p < 0.001], were more often under mechanical ventilation [41/97 (42%) vs. 2/53 (4%), p < 0.001] and had more frequently lymphopenia [57/97 (59%) vs. 23/53 (43%), p = 0.07)], anemia [50/97 (51.5%) vs. 14/53 (26%), p = 0.003], and hypoalbuminemia [55/97 (58%) vs. 22/53 (42%), p = 0.08] (Table 3).

**Table 3:** Baseline and clinical characteristics of COVID-19 patients between the Indeterminate vs determinate QFT®-Plus test.

	COVID-19 N = 150 (%)	Indeterminate n = 97(%)	Determinate n = 53(%)	Bivariate p value
Male	104(69)	70(72)	34(64)	0.30
Age*	58(48-68)	61(48-72)	54(48-64)	0.02
Obesity	96(64)	26(54)	70(69)	0.07
Pharmacological immunosuppression	74(49)	50(51.5)	24(45)	0.46
Rheumatic disease	16(11)	9(9)	7(13)	0.45
Chronic kidney disease	9(6)	5(5)	4(7.5)	0.72
Hematological malignancy	4(3)	1(1)	3(6)	0.127
Solid neoplasm	4(3)	4(4)	0	0.29
Days since start COVID-19 and sample test*	7(4–12)	9(6–14)	4(2–7)	<0.001
Corticosteroids for COVID-19	98(65)	68(70)	30(57)	0.10
Mechanical ventilation	43(29)	41(42)	2(4)	<0.001
Lymphopenia	80(53)	57(59)	23(43)	0.07
Severe Lymphopenia	29(19)	23(24)	6(11)	0.07
Anemia	64(43)	50(51.5)	14(26)	0.003
Higher CRP	143(95)	92(95)	51(96)	0.70
Hypoalbuminemia	77(52)	55(58)	22(42)	0.08

Note: CRP, C-reactive protein. *Data are presented in median with interquartile range (IQR).

### Second QFT®-Plus test

Among patients with indeterminate results, only 37% (n = 129/352) underwent a second or repeat QFT®-Plus test. Of these, 43% (n = 55/129) were tested within 30 days, while 57% (n = 74/129) were tested after 30 days. Among the repeat tests, 32.5% (n = 42/129) remained indeterminate, 62% (n = 80/129) were negative, and 5% (n = 7/129) were positive.

Lymphopenia [76% (n = 32/42), p < 0.01] and anemia [81% (n = 34/42), p < 0.01] were significantly associated with a second indeterminate result. Notably, 67% (n = 28/42) of indeterminate results occurred during hospitalization, and 82% (n = 23/28) were tested within 30 days of the initial test (p < 0.01).

In the COVID-19 patients, only 18.5% (n = 18/97) had a second test, and 72% (n = 13/18) before 30 days, of which 28% (n = 5/18) remained indeterminate, 67% (n = 12/18) were negative, and 5% (n = 1/18) positive.

## Discussion

Our study identified a high prevalence of indeterminate results in the COVID-19 population. Ward et al. [20] previously reported this finding, documenting an increase in indeterminate results from 8.7% to 15.5% in North Carolina. Similarly, other studies have observed up to 35% increases in Spain [16] and Italy [17].

The underlying mechanism behind most of our indeterminate test results was a low mitogen response, indicating that the T cells of affected subjects had an insufficient number or functional TCD4+ and TCD8 + cells to produce IFN-γ in response to the ESAT-6 and CFP-10 antigens contained in the Tb1 and Tb2 test tubes. Additionally, these subjects exhibited low IFN-γ production in response to the universal mitogen phytohemagglutinin. Once their general immune state was restored, their sera responded to the mitogen while maintaining an absence of response to *Mycobacterium tuberculosis* antigens, leading to negative test results [21].

Palacios-Gutiérrez et al. reported that 90% of patients with an initial indeterminate result who underwent repeat testing had a negative result [22]. This finding reinforces that an indeterminate result should not be interpreted as positive; patients with additional evidence of TBI should have timely treatment, regardless of their QuantiFERON result [23].

Corticosteroids have previously been implicated as a cause of indeterminate results; however, our study did not find such an association. Notably, we were able to analyze patients both with and without concurrent steroid use, as corticosteroids were not yet considered standard of care at the time. The generalized immune activation observed in severe COVID-19 may contribute to indeterminate results. Ward et al. [20] reported a significant reduction in IFN-γ production (1.29 IU/mL) after mitogen stimulation in COVID-19 patients compared to healthy controls (7.87 IU/mL). Furthermore, increased levels of IL-6 and IL-10 correlate with COVID-19 severity, and the dysregulated Th1 immune response in critically ill patients reinforces this theory [20,24]. Our study found that COVID-19 was independently associated with indeterminate QFT-TB Gold test results. Evidence suggests that as patients recover from COVID-19, IFN-γ production may return to normal [18]. Consequently, follow-up testing is advisable for individuals with indeterminate QFT results. In our study, most follow-up tests yielded determinate results; however, there are currently no established guidelines regarding the optimal timing for retesting.

Corticosteroids became standard in severe COVID-19 cases following the preliminary results of the RECOVERY trial in June 2020, which demonstrated reduced mortality [25]. Nearly all COVID-19 patients in our study underwent QFT testing during hospitalization. While not a mandatory practice, QFT testing was performed in cases requiring high doses of steroids or tocilizumab. Interestingly, 70% of COVID-19 patients who received dexamethasone had indeterminate QFT results; however, they did not have statistical significance, unlike some studies mentioned by Tekaya et al. [18]. The impact of glucocorticoids on IFN-γ release has been attributed to their suppressive effects on T-cell function, potentially affecting test interpretation. In addition to T-cell suppression, glucocorticoids induce apoptosis and inhibit Th1-derived cytokines, including IFN-γ, IL-2, IL-10, and TNF-α, which may influence results [26,27]. Although the dexamethasone dosage used in COVID-19 treatment is equivalent to high-dose prednisone (>30 mg), previous studies have reported that prednisone doses >40 mg are associated with indeterminate QFT results [28]. Notably, the study by Bélard et al. had a small sample size, so its findings should be interpreted with caution and not be used for clinical decision-making. Additionally, our results align with other studies that found no correlation between corticosteroid dose and indeterminate QFT results [13,27]. Furthermore, COVID-19 patients with an indeterminate QFT may have been more seriously ill or had received treatments at the time of sample collection that could have altered the result.

Regarding other factors associated with indeterminate results of QFT®-Plus, advanced age has been implicated [29], likely due to reduced interferon production and immunosenescence in older adults [11]. However, other studies, including ours, have not found a consistent association between advanced age and indeterminate results except in the COVID-19 subgroup [30,31]. COVID-19 infection has been associated with greater severity and worse outcomes in older patients, which could be correlated with the findings of older age associated with indeterminate QFT-Plus in the subgroup of COVID-19 patients.

Our analysis identified an association between lymphopenia and indeterminate QFT-Plus results, consistent with findings from Jung et al., who reported an odds ratio (OR) of 6.28 (95% CI: 2.64–14.92, p < 0.001) for lymphopenia as a predictor of indeterminate results [30]. The literature suggests that lymphopenia reduces IFN-γ levels, leading to indeterminate results, a phenomenon also observed in chronic diseases such as HIV and hematologic malignancies [32,33]. In our study, total lymphocyte count was significantly lower in the indeterminate group, and severe lymphopenia remained statistically significant in the multivariate analysis. This is particularly relevant, as low lymphocyte counts reduce mitogen response, limiting the likelihood of obtaining a determinate result [11,12]. In addition, lymphopenia in COVID-19 has also been reported to be associated with indeterminate QFT [18].

As expected, pharmacological immunosuppression at the time of testing was associated with indeterminate QFT-Plus results, consistent with previous reports on immunosuppressive therapies such as sulfasalazine, TNF-α inhibitors, and corticosteroids, regardless of dose. One proposed mechanism is that immunosuppressants may interfere with IGRA test mitogen production through mechanisms that inhibit IFN-γ activity [34]. Our study included patients receiving various immunosuppressive drugs beyond corticosteroids, including rituximab, tacrolimus, and mycophenolate.

Hospitalized patients often exhibit a hypercatabolic state, fluid shifts to the third space, and multiple disruptions in cellular pathways, which may impair immune activation and contribute to indeterminate test results. Among non-COVID-19 hospitalized patients, the primary reasons for admission were heterogeneous; however, the presence of lymphopenia, anemia, hypoalbuminemia, and elevated CRP in patients with indeterminate QFT-Plus results reflected the severity of their illness. These patients likely had multiple risk factors, as described in previous cohorts where up to 61% of critically ill patients with high inflammatory markers had indeterminate QFT results [31,35,36].

Although representing a small subgroup, low BMI and chronic liver or kidney disease were associated with indeterminate QFT results in the bivariate analysis. This is likely due to malnutrition and immune dysregulation in these conditions, though we cannot exclude the possibility of confounding factors [37].

The largest U.S. cohort evaluating QFT results analyzed over two million tests, including more than 300,000 indeterminate results (4.2%) [19]. Among patients who underwent repeat testing, 50% converted to a negative result within 30 days, and 66.8% converted after 30 days, suggesting that unstable immune response and/or assay-related variability at the time of testing play a significant role. However, 36% and 23% of patients remained indeterminate upon repeat testing, a finding consistent with our results.

Our study has several limitations, including its retrospective design and the inability to perform repeat testing on all indeterminate cases. However, a key strength is including a large, diverse patient population, including individuals with COVID-19, making our findings more applicable to real-world clinical practice.

Screening for TBI before initiating immunosuppressive therapy is a best practice. However, our study demonstrates that despite advances in QFT methodology, patients with severe COVID-19 and hospitalized individuals with lymphopenia, anemia, or immunosuppressive treatment may have indeterminate test results. These patients should be retested once their clinical condition improves. Ideally, screening should occur during disease stability or before initiating immunosuppression to minimize the risk of indeterminate results and avoid delaying treatment, particularly in resource-limited settings. Given the lack of standardized guidelines for managing indeterminate QFT®-Plus results, particularly regarding the optimal timing for retesting, our findings underscore the need for clinical protocols to guide decision-making in high-risk or critically ill patients.

## Conclusion

A high frequency of indeterminate QFT®-Plus results was observed in patients with severe COVID-19. Overall, indeterminate QFT®-Plus results were associated with markers of critical illness, including severe lymphopenia and anemia, as well as with immunosuppression and hospitalization at the time of testing. Our findings emphasize the importance of diagnostic test stewardship and selecting the appropriate timing for testing, as QFT®-Plus remains the most effective screening tool for tuberculosis infection.
